# Unveiling synergistic antioxidant combinations for α-tocopherol in emulsions: A spectrophotometric-mathematical approach

**DOI:** 10.1016/j.crfs.2025.101134

**Published:** 2025-07-02

**Authors:** Camille Robichon, Pierre Villeneuve, Philippe Bohuon, Bruno Baréa, Nathalie Barouh, Francis Courtois, Frédéric Fine, Erwann Durand

**Affiliations:** a CIRAD, UMR QualiSud, F-34398, Montpellier, France; b QUALISUD, Univ Montpellier, Avignon Université, CIRAD, Institut Agro, IRD, Université de La Réunion, Montpellier, France; c TERRES INOVIA, Parc Industriel – 11 Rue Monge, Pessac, 33600, France

**Keywords:** α-Tocopherol, Antioxidants, Synergy, Lipid oxidation, Emulsion, Modeling, Carnosic acid, Dieckol

## Abstract

This study presents a novel method based on a spectrophotometric assay coupled with mathematical modeling (Weibull interactions modeling CAT) to evaluate synergistic interactions of antioxidants in an oil-in-water model emulsion. The approach addresses a major limitation in current assays by eliminating interference from pro-/antioxidant pathways induced by exogenous radical initiators or buffers, allowing for a more accurate assessment of antioxidant efficacy. Using the Weibull equation, key kinetic parameters (LagP, α, β) were extracted to characterize the impact of antioxidant combinations on lipid oxidation. Results showed that certain combinations, such as α-tocopherol with carnosic acid, dieckol, and myricetin, exhibited concentration-dependent synergistic effects. Among these, carnosic acid in equimolar ratios demonstrated particularly promising interactions. In contrast, ascorbic acid revealed antagonistic behaviour when combined with α-tocopherol. Additionally, the presence of ferrous ions significantly influenced antioxidant interactions, highlighting their role in modulating underlying mechanisms. Overall, carnosic acid and dieckol emerged as strong candidates for use in combination with α-tocopherol under specific conditions. This method provides a refined approach for screening antioxidant synergies and contributes to the development of more effective antioxidant formulations in emulsified systems.

## Highlights

• Spectrophotometric assay with Weibull modelling identifies key oxidation parameters.
• α-tocopherol concentration affects its combination efficiency with other antioxidants.
• Synergy between α-tocopherol and carnosic acid, dieckol, and myricetin is ratio-dependent.
• This method allows to determine optimal conditions for α-tocopherol antioxidant synergy.

## Graphical abstract

**Figure:**
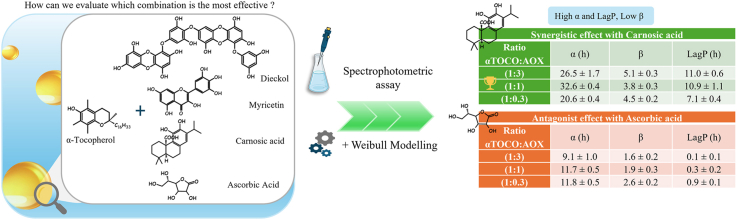

## 1 Introduction

Lipid oxidation is a complex phenomenon that significantly affects the quality of food products, leading to off-flavours and rancidity, the degradation of fat-soluble vitamins (e.g., A, E) and unsaturated fatty acids, and the formation of harmful oxidation products (Schaich, 2024; Shahidi and Hossain, 2022). In food products, lipid oxidation depends on several factors including endogenous or added antioxidants, the presence of prooxidants, the processing and storage conditions (Shahidi and Hossain, 2022; Vieira et al., 2017). This complex phenomenon may occur through three different pathways: (i) nonenzymatic free radical chain autoxidation, (ii) nonradical photooxidation, and (iii) enzymatic oxidation primarily mediated by lipoxygenases (Hennebelle et al., 2024). The latter two mechanisms can be controlled during the production phase through appropriate treatments and the choice of suitable packaging. However, autoxidation remains a major challenge for manufacturers and consumers alike. Autoxidation is a radical propagation reaction which can proceed via hydroperoxide-independent or dependent induced mechanisms affected by heat, light, metals, or other catalysts (Durand et al., 2025). In addition, these mechanisms are significantly influenced by the physical structure of the system (e.g., bulk oils, emulsions, liposomal formulations) (Decker et al., 2017; Hennebelle et al., 2024).

One effective strategy to mitigate lipid oxidation is adding antioxidants that extend the lag phase of lipid oxidation. Recently, research on natural antioxidants has intensified including phenolic compounds (flavonoids, tannin, tocopherols, and tocotrienols) or carotenoids (Laguerre et al., 2007). Among these, tocopherols, particularly α-tocopherol (αTOH), have been extensively studied since their discovery and isolation in the early 20th century (Azzi, 2018; Evans and Bishop, 1922). Tocopherols act as radical scavengers by donating hydrogen or chelating metal ions (Barouh et al., 2022). Given its widespread presence in nature and its important role in oxidative defense, α-tocopherol (αTOH) is a logical candidate for enhancing antioxidant activity in food products (Delgado et al., 2020). The literature already highlights several potential synergistic effects with other natural antioxidants that could further improve its efficacy (Decker et al., 2017; Bayram et al., 2023).

Indeed, synergistic interactions between antioxidants offer several advantages, as they maximise their effect and extend the shelf life of products (Bayram and Decker, 2023). However, understanding the mechanisms behind these synergistic effects remains challenging. It is suggested that they may result from different mechanisms: (i) regeneration of the stronger antioxidant by the weaker one, (ii) the combined effects of free radical scavenging and metal chelation, which enhance the antioxidant's scope, or (iii) a varied distribution of antioxidants within the food matrix, where their positioning can influence their effectiveness. For example, Bayram et al. (Bayram and Decker, 2024) demonstrated the capacity of myricetin to regenerate tocopherols in oil-in-water emulsions. Similarly, Tang et al. (2023) evaluated the beneficial combination of phospholipids and tocopherols highlighting the potential for tocopherols to be regenerated by phospholipids. Zhang et al. (2024) also studied this phenomenon with the interactions between chlorogenic acid and tocopherols. Concerning the impact of antioxidant distribution, Tang et al. highlighted the role of phospholipids in the distribution of tocopherols at the interface (Tang et al., 2023). However, these results are specific to a certain organisation and environmental conditions, making them difficult to generalise to all systems. As an example, Becker et al. (2007) observed that quercetin exhibited strong synergy with αTOH in emulsion but an antagonistic effect in bulk oil. The hypothesis behind this difference is complex, but it may be attributed to variations in antioxidant partitioning in the different systems (Becker et al., 2007). Additionally, the pH and the presence of other compounds (metals, other antioxidants, proteins) can also modulate synergistic interactions as demonstrated by Kittipongpittaya et al. (2016) who found that rosmarinic acid and αTOH exhibit synergy at neutral pH but only an additive effect at lower pH. These examples reveal the importance of contextualising the interaction between two antioxidants to better identify the key parameters governing their synergistic effect (Gordon and Decker, 2010).

Consequently, establishing an efficient *in vitro* method to accurately evaluate interactions between antioxidants in lipid oxidation is essential. Today, various direct and indirect methods are used to assess antioxidant efficiency (AE). Although the simplest, indirect methods are not suitable for this purpose. These methods involve removing a non-oxidizable element and assessing a compound's ability to reduce a stable artificial free radical. Since there is no competitive reaction between the candidate antioxidant and the oxidizing substrate, these methods are commonly referred to as non-competitive methods with the DPPH assay being the most well-known example. The results obtained from these methods provide only an indication of “radical trapping power” or “reducing power” rather than true antioxidant activity and therefore cannot be used to assess synergistic activities (Amorati and Valgimigli, 2015). Moreover, these indirect methods fail to address how environmental factors influence AE. An alternative to overcome these limitations is the use of direct methods which monitor the oxidation of species either by tracking their disappearance or by measuring the formation of their oxidation products, such as primary and/or secondary oxidation products. However, this second approach can be challenging due to the instability and diversity of these compounds. To ensure reliable trends, both types of oxidation products must be evaluated, which significantly increases the experimental workload (Laguerre et al., 2007). In this context, Laguerre et al. (2008) developed a direct method (CAT assay) to evaluate the AE. Based on tung oil, which is rich in trieleostearin, this test follows the oxidation of α-eleostearic acid when exposed to oxidizing conditions induced by an azo-initiator. The conjugated triene structure of eleostearic acid can be easily tracked by monitoring the loss of absorbance at λ = 273, in both the presence and absence of a given antioxidant (Panya et al., 2015). However, the oxidizing conditions used in the assay may influence the antioxidant's mechanisms. This issue is particularly pronounced in synergistic antioxidant interactions, where a dominant and preferential oxidation pathway may prevail, potentially leading to biased conclusions (Phonsatta et al., 2020). Indeed, in many studies where lipid oxidation kinetics or antioxidant efficacies are measured, chemical pathways are artificially accelerated using chemical azo initiators. However, the addition of such initiators may introduce significant biases as these artificial conditions do not accurately replicate natural mechanisms, leading to diverse oxidation products with varying polarities and multiple oxidation pathways. In addition, lipid oxidation in emulsions is also often studied using buffer solutions as the continuous phase. While this approach is understandable to maintain stable pH conditions throughout study, the use of buffers can modify oxidation kinetics. Buffers can change the charge and composition of the lipid interface and the formation of metal-buffer complexes can modify pro- and antioxidant mechanisms by affecting redox potential or metal distribution. This, in turn, can impact hydroperoxide reduction or oxidation, radical scavenging, and related reactions (Daoud et al., 2021). As a result, the often-underestimated impact of buffers may lead to misleading conclusions when evaluating AE in model systems, compared to their behaviours in real food.

In this study, we developed a method based on the CAT, intending to minimize the biases associated with the use of azo initiator and buffer solutions. Once optimized, the method was used to identify the optimal synergistic effects of combinations of αTOH with other antioxidants. The resulting kinetics were described using a Weibull equation which is a suitable model to describe degradation reactions, including oxidation (Barbieri et al., 2020; Bayram et al., 2024). This model enabled us to calculate a combination factor (CF) which, correlated with the AE (described by the lag phase), allows characterization of the interaction between the two antioxidants. The antioxidants tested alongside αTOH (carnosic acid (CA), myricetin (Myr), dieckol (Dl), and ascorbic acid (AA)) were selected for their structural, reactive, and solubility diversity, leading to different distributions within the emulsion and varied reactivities with αTOH. Furthermore, a kinetic model was developed to describe the absorbance loss during the oxidation reaction and to characterize the synergistic effects of αTOH combinations with antioxidants.

## 2 Materials and methods

### 2.1 Chemicals

Tung oil from *Aleurites for*dii seeds (tung oil, average MW = 872 g/mol, Peroxide Value < 1.5 meq O_2_/kg, Iron content <0.9 mg/kg), myricetin (≥96 %), ascorbic acid (≥98 %), dieckol (≥90 %), carnosic acid (≥99 %), αTOH (≥97 %), tocopherol standards, phosphate buffer solution-pH 7.2 (PBS), polyoxyethylene(23)lauryl ether (Brij 35, estimated MW = 1199 g/mol), iron chloride (II) were purchased from Sigma (Saint Quentin, France). All solvents used were HPLC or analytical grade and were purchased from Sigma (Saint Quentin, France).

### 2.2 Measurement of peroxide value

The Peroxide value (PV) of tung oil was determined by iodometric titration in accordance with the international norm ISO 3960. Briefly, a known quantity of oil (0.5–2 g) was dissolved in chloroform (10 mL) in an around-bottom flask, followed by the addition of acetic acid (15 mL). To initiate the reaction, a freshly prepared potassium iodide (1 mL) was added. The flask was immediately sealed and stirred for 1 min. After allowing the reaction to proceed in the dark for 5 min, it was quenched by the addition of 75 mL of distilled water. The manual titration was made with a sodium thiosulfate solution (0.002 N) with iodine as an indicator, and the HC was determined as follows:

(1) $$PV=\frac{\left(V-V0\right)\times N\times 1000}{m}$$

with *V*, the volume (mL) of the sodium thiosulfate solution in the presence of the sample. *V*0 is the volume (mL) of the sodium thiosulfate solution in the absence of the sample, *m* is the mass of the sample (g), and *N* means the normality of the sodium thiosulfate solution. The results obtained using this method were expressed in meqO_2_/Kg of oil.

### 2.3 Stripping of tung oil

Tung oil was stripped of its polar compounds, including tocopherols, by passing 25 mL of a hexane solution of tung oil (200 mg/mL) through a Redisep column prefilled with alumina (Cat: 19.997–4, 50g), connected to a Reveleris X2 flash chromatography system (Buchi, Villebon sur Yvette, France). The solution was eluted with hexane, and fractions were selected according to HPLC analysis. Complete removal of tocopherols was checked by HPLC (see Section Quantification of tocopherols via HPLC-Fluorescence). Subsequently, hexane was evaporated under vacuum (∼20 kPa) at 30 °C using a rotatory evaporator equipped with a vacuum pump (Laborport, KIF Neuberger GmbH, Freiburg, Germany). The stripped tung oil was then aliquoted into brown glass tube (4 mL) and residual solvents were removed by bubbling with a stream of nitrogen.

### 2.4 Determination of Total Fatty Acid (TFA) profiles of tung oil

The TFA profile of tung oil was determined by gas chromatography (GC) following the NF T60-233 standard method with slight modifications. Briefly, sodium methylate solution (1 mL) containing phenolphthalein was added to oil sample (15 mg). After heating at 65 °C for 10 min, hydrochloric methanol (1 mL) was added and the mixture was reheated at 65 °C for another 10 min. After cooling, hexane (1 mL) and water (1 mL) were added. The mixture was centrifuged at 1500 rpm for 5 min using a Hettich Rotina 380R centrifuge (Hettich, Tuttlingen, Germany), and the organic phase was collected for GC analysis. GC analysis was performed using a Focus GC (Thermo Electron Corporation, Massachusetts, USA), equipped with a split injector (ratio of 1/20), a CP-Cil 88 Varian capillary column (50 m × 0.25 mm with 0.2 μm film thickness; Agilent Chrompack), and helium (1 mL min^−1^) as carrier gas. Fatty acid methyl esters (FAME) were analysed by a flame ionization detector and ChromCard software data system (version 2005, Thermo FisherScientific, Massachusetts, USA). The column temperature was initially set at 150 °C, increased to 225 °C at a rate of 5 °C.min^−1^ and held at 225 °C for 10 min. The injector and detector temperatures were set at 250 and 270 °C, respectively. FAME were identified by comparing retention times with those of an external FAME standard mixture (mix37 EMAG, Supelco). The fatty acid composition was as follows:C16:0, 1.9 %; C18:0, 2.1 %; C18:1n9c, 4.9 %; C18:1n7, 0.2 %; C18:2n6c, 6.7 %; C20:1, 0.7 %; C18:3 (α-eleostearic acid), 79.8 %; C18:3 (β-eleostearic acid), 3.6 %)

### 2.5 Quantification of tocopherols by HPLC-fluorescence

Tocopherols content in tung oil were quantified using oil samples (10 mg) diluted in hexane (10 mg/mL). Four distinct tocopherols isoforms (α, β, γ and δ) were quantified according to the ISO-FDIS 9936 standard. HPLC analysis was carried out using an Ultimate 3000 equipped with a fluorescence detector FL 3000 (Thermo electron, Massachusetts, USA) and an ACE silica column 250 mm × 4.6 mm, 5 μm (A.I.T. France, Corneilles en Parisis, France). The mobile phase consisted of hexane/1,4 dioxane (97:3 v/v) at a flow rate of 1.3 mL min^−1^. The column temperature was maintained at 27 °C. Fluorescence detection was set at 290 and 330 nm for excitation and emission, respectively. The injection volume was 100 μL. Quantification was performed using calibration curves prepared from standard solutions of each tocopherol isoform (reference 613424).

### 2.6 Incorporation of α-tocopherol in tung oil

An ethanol solution of αTOH was prepared at a concentration of approximately 1.21 g. L^−1^. A 1 mL aliquot of this solution was evaporated under a stream of nitrogen, after which 800 μL of stripped tung oil was added. The resulting αTOH-enriched oil was then diluted with additional stripped tung oil to achieve two final αTOH concentrations: 1.21 g. L^−1^ (1140 ppm, 2.8 mM) and 0.4 g. L^−1^ (380 ppm, 0.93 mM) respectively.

### 2.7 Preparation of tung oil-in-water nanoemulsions

In a 250-mL Erlenmeyer flask, a Brij 35 solution (150 mL, 19.8 mg. L^−1^) was combined with tung oil (32 μL) (with or without αTOH) and pre-mixed by vortexing for 30 s. The mixture was then homogenized using a Silverson L5M-A (Silverson, Silverson France) at 10,000 rpm for 3 min. To standardize the incorporation of antioxidants, regardless of their lipophilic or hydrophilic nature, the second antioxidant was added post-emulsification. Antioxidants were introduced via ethanol solutions without an intermediate stabilization phase. The final concentrations of αTOH in the nanoemulsion 0.2 μM and 0.6 μM, corresponding to 380 ppm and 1140 ppm, respectively, in the tung oil. Molar ratios of αTOH to the second antioxidant were adjusted to 1:3, 1:1 and 1:0.3.

### 2.8 Monitoring oxidation in tung oil-in-water nanoemulsion

A 96-well microplate (Greiner) was filled with nanoemulsion (115 μL per well) and brought to a final volume of 250 μL with an aqueous solution. For experiments involving ferrous ion initiated-oxidation, a 2.5 μM FeCl_2_ solution (10 μL) was added to each well and the volume was adjusted to and completed with an aqueous solution to obtain 250 μL with the same aqueous solution. The microplate was placed a humidity-controlled chamber within a SPARK 10M microplate reader (Tecan, Grödig, Austria). An initial absorbance reading at 234 nm was taken to detect conjugated dienes and to verify the oxidation state of the oil prior to kinetic measurements. Subsequent absorbance readings were recorded at 273 nm every 10 min for the first 2 h, then hourly until all samples reached a plateau indicating the end of oxidation.

### 2.9 Particle size measurement using Dynamic Light Scattering (DLS)

The droplet size distribution of the nanoemulsions was determined by laser light scattering using a Mastersizer 3000 laser diffractometer (Malvern Instruments, Malvern, UK). Measurements were conducted at 25 °C on freshly prepared nanoemulsions (day 0) and after four days of storage (day 4) to assess oil droplet stability. Samples were diluted tenfold in distilled water previously filtered through a 0.2 μm RC filter. The refractive index of tung oil and water at 25 °C was taken as 1.47 and 1.33, respectively. All measurements were performed in triplicate and results are reported as Z-average (μm) and polydispersity index (PI).

### 2.10 Statistical analysis

Each kinetic experiment was carried out on emulsion triplicates. Results are presented as mean values mean values ± standard deviations. Different tests were performed according to the relationship between samples and group characteristics to determine which mean values were different (significance level was set at p < 0.05): One-way ANOVA calculations included sum of squares (SSB, SSW) and F statistic, with Tukey's HSD post-hoc test. Paired data were analysed using a paired *t*-test. Independent groups were analysed using an independent *t*-test. Statistical analyses were performed in MATLAB using user-defined means, standard deviations and sample sizes to simulate the data.

### 2.11 Kinetic oxidation model development

The oxidation kinetic model was developed to characterize and quantify the effects of antioxidants, including additives and synergy interactions, on the oxidation kinetics of α-eleostearic acid within a nanoemulsion. Modeling the lipid oxidation curves provides deeper insights into the oxidation dynamics of the system and enables direct comparisons across different formulations (Goujot et al., 2019; Schroën and Berton-Carabin, 2022). In this kinetic model, absorbance at time *t*, denoted A, was used as the response variable. This response was dimensionless, $A^{*}$, and ranges from 1 to 0, according to:

(2) $$A^{*}=\frac{A-A_{\infty }}{A_{0}-A_{\infty }}$$

Where $A_{0}$ and $A_{\infty }$ were the initial and infinite-time absorbance values, respectively.

#### 2.11.1 Weibull distribution model

This decrease in absorbance during oxidation was modeled using the Weibull equation. Due to its flexibility, the Weibull distribution has also been applied in life data analysis (van Boekel, 2002), and has recently been used to describe lipid oxidation in biological systems (Bayram et al., 2024; Parra-Escudero et al., 2025). While the conventional first-order kinetic model assumes homogeneity in lipid oxidation behavior within nanoemulsions, our approach considers the heterogeneity arising from variations in oil droplet size and the oxidative resistance of individual droplets. These variations (introduced by commonly used homogenization techniques such as the Silverson) are assumed to follow a Weibull distribution. Consequently, the oxidation kinetics were described using a Weibull-based model to better capture this intrinsic variability.

(3) $$A^{*}=\exp\left[-{\left(\frac{t}{\alpha }\right)}^{\beta }\right]when\;t\;>\;LagP$$

LagP is the duration of the lag phase during which the antioxidant prevents radical chain formation, characterized by a reduced rate of oxidation. Throughout this period, t≤LagP, $A^{*}$ remains constant at 1. The Weibull distribution is defined by two parameters:

• α>0, is the scale parameter which represents a characteristic time, influencing the temporal positioning of the degradation curve. Regardless of the shape parameter β, when t=α, the absorbance decreases by 63 %, indicating the characteristic time at which a significant fraction of the antioxidant capacity has degraded.
• β>0, is the dimensionless shape parameter influences the shape of the curve through its slope and mode of evolution. First-order kinetics reaction is a particular case of Eq. (3), where β=1, making the reaction rate constant simply 1/α ($h^{-1}$). For β>1, degradation accelerates over time, typically due to catalytic effects or auto-accelerated mechanisms enhancing the reaction rate as degradation progresses. As β tends to infinity the function converges to a Dirac delta function centred at α.

#### 2.11.2 Kinetic parameter estimation and statistical methods

The two Weibull parameters (α and β) were determined using the Curve Fitting Toolbox 3.6 in MATLAB® (The MathWorks Inc., Natick, MA, USA) by applying the NonlinearLeastSquares method. The quality of the model fit was evaluated using the root mean square error (*RMSE*) of the reduced absorbance and the adjusted *R*^2^. Confidence intervals for both parameters (IC_α and IC_β) were calculated at the 95 % confidence level. The *LagP* was subsequently identified as the time at which the probability density function, PDF (as described in equation (4)) reaches a threshold value of 0.005. This condition is expressed in Equation (5).

(4) $$\frac{dA^{*}}{dt}=-\frac{\beta }{\alpha }{\left(\frac{t}{\alpha }\right)}^{\beta -1}\;\exp\left[-{\left(\frac{t}{\alpha }\right)}^{\beta }\right]=-\text{PDF}$$

(5) $$PDF\left(t=LagP\right)=5\times 10^{-3}\;\left(h^{-1}\right)$$

The confidence interval for *LagP* was determined using the Monte Carlo simulation method (Hessler, 1997), propagating the uncertainties of *α* and *β* based on their respective confidence intervals over 10,000 iterations.

## 3 Results and discussion

α-eleostearic acid was identified as the predominant fatty acid (∼75 % w/w) in tung oil. The stripping process. The stripping process did not significantly alter the overall fatty acid composition (Section 2.4). Additionally, the peroxide value of tung oil was measured at 1.5 ± 0.3 meqO_2_/kg of oil. The maximum absorbance (λ max) at 273 nm for tung oil, when formulated as an oil-in-water emulsion, reached a raw absorbance value of approximately 1.7. This measurement may serve, alongside DLS analysis, as an indicator of proper nanoemulsion formation. Early-stage oil degradation was assessed by measuring absorbance at λ = 234 nm (see section 2.8), with values expected to remain below 0.3. DLS analysis performed immediately after emulsion preparation (t = 0) revealed an average droplet size of 196.5 ± 16.8 nm (PI = 0.3). This stability was maintained over a 4 day-period, with the droplet size remaining at 182.3 ± 7.3 nm (PI = 0.3), confirming the long-term stability of the nanoemulsion throughout the study.

### 3.1 Evaluation of the effect of buffer type and concentration on oxidation kinetics

**Fig. 1:**
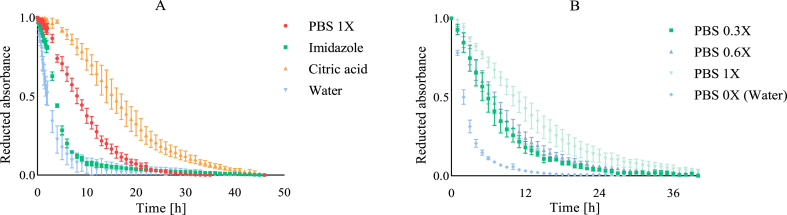
Effect of buffer type (10 mM adjusted to pH 7) (A) and effect of different concentrations of PBS with X = 10 mM (B) on oxidation kinetic. Data are presented as mean values ± standard deviations (n = 3).

The initial version of the CAT assay was developed using a phosphate buffer (PBS, pH 7, 10 mM) to maintain stable pH conditions throughout the kinetic measurements (Laguerre et al., 2008). Indeed, pH fluctuations in aqueous systems can influence the ionization state of molecules, their solubility, their distribution and the overall physicochemical organisation of the emulsion, which in turn may alter oxidation mechanisms. However, the influence of buffer chemical structure on oxidation kinetics remains less explored particularly in relation to the formation of buffer-metal complexes that can modulate pro/antioxidant mechanisms such as hydroperoxide decomposition and radical scavenging. Previous studies by Daoud et al. (2021) and previously Yoshimura et al. (1992) demonstrated that phosphate and citrate buffers can chelate metal ions like iron, reducing their pro-oxidant activity*.* In light of these findings and to avoid potential interference from buffers when evaluating synergistic interactions between antioxidants, we first assessed how the nature of the continuous phase affects oxidation kinetics. To this end, water and three commonly used buffers (PBS, imidazole, and citric acid), all at pH 7, were evaluated (Fig. 1A). Results revealed that that all buffers, irrespective of type, slowed oxidation kinetics compared to the non-buffered aqueous solution. Among them, citric acid exerted the greatest inhibitory effect, while imidazole had the least. These differences are likely due to their chemical structures, particularly the number of electron-donating groups, which may influence interactions with iron or other reactive species. Furthermore, buffer concentration also significantly impacted oxidation kinetics. Increasing the concentration of phosphate buffer (PBS) markedly reduced oxidation (Fig. 1B). Consequently, to eliminate potential buffer-related interference when evaluating antioxidant mechanisms, this model assay was further designed without any pH-buffering agent. This approach minimizes the variables specific to each system and allows for a clearer investigation of physicochemical mechanisms underlying antioxidant synergy.

### 3.2 Weibull modeling for antioxidant efficiency assessment

**Fig. 2:**
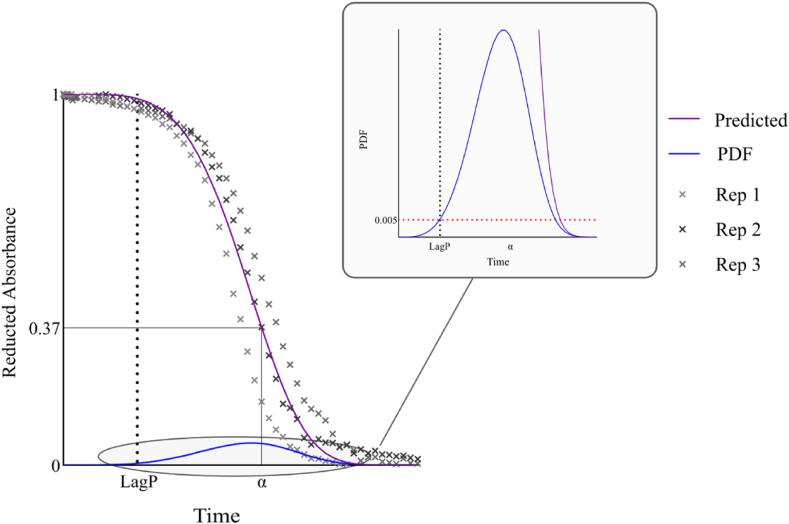
Experimental reduced oxidation kinetics obtained (Replicates 1–3) and fitted Weibull model: predicted kinetic (Equation (3)), PDF (first derivative (Equation (4)),), Threshold ((Equation (5)), red dotted line) and LagP (black dotted line).

The kinetic measurements were conducted in three replicates for each experimental condition, and the resulting datasets were fitted using the Weibull kinetic model. The model provided a good fit across all conditions, as indicated by low root mean square error (RMSE) values and high coefficients of determination (R^2^), consistent with previously reported findings (Barbieri et al., 2020) (Fig. 2 and Supplementary Data).

The Weibull-like analysis facilitates the determination of LagP by evaluating the first derivative (**see section 2.11.2**). Once the LagP of the individual antioxidants (αTOH and the second antioxidant (AOX)) as well as their combination (αTOH‐AOX) are determined, the effectiveness of the antioxidant combination is quantified through the combination factor (CF) describing how the two antioxidants interact. This factor is defined as the ratio of the experimentally obtained LagP to the additive LagP.

(6) $$CF=\frac{LagP\left(\alpha TOH‐AOX\right)}{LagP\left(\alpha \text{TOH}\right)+LagP\left(AOX\right)}$$

**Fig. 3:**
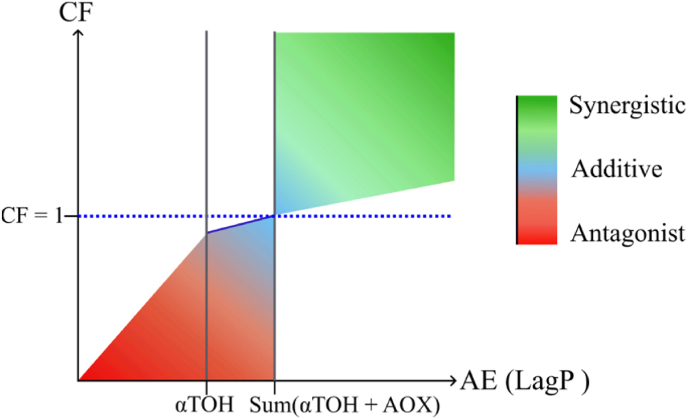
Graphical representation of combination factor (CF) and antioxidant efficiency (AE) derived from calculated LagP values. Colors denote the type of response in the combination of αTOH and a second antioxidant (AOX): synergistic (green), additive (blue), or antagonistic (red). Color intensity reflects the magnitude of the effect, with higher intensity indicating a stronger effect.

The nature of the interaction between the two antioxidants is determined by the CF: CF > 1 indicates synergy, CF < 1 suggests antagonism, and CF = 1 corresponds to an additive effect. Although related, the CF should not be confused with antioxidant efficiency (AE), which refers to the absolute LagP value of the antioxidants, either alone or in combination. Therefore, the evaluation of antioxidant combinations requires consideration of both the CF and AE values. By correlating CF with AE (based on LagP), distinct phases emerge that clearly indicate the effectiveness of the antioxidant combination (Fig. 3). The slope (dark blue line) depends on the difference between the LagP of αTOH alone and the additive LagP of αTOH + AOX. The CF must be seen as the result of the difference in the intensity of all antioxidants physicochemical mechanisms and reactivities versus the prooxidant mechanisms and reactivities, whether they act independently or interdependently.

The kinetic analysis of the data underscores the relevance of using a two-parameter Weibull model with α and β, incorporating LagP. Statistical analysis showed a strong positive correlation between these two parameters (r = 0.94, p < 0.0005), indicating that a longer LagP corresponds to a greater characteristic time α, reflective of slower oxidation process. This correlation suggests that mechanisms delaying oxidation not only affect its initiation but also its subsequent progression. LagP represents the induction period prior to significant oxidation onset, and its importance justifies using a Weibull model that explicitly incorporates this parameter. Both α and LagP are highly sensitive to oxidation conditions; their values decrease significantly under metal-induced oxidation, indicating accelerated onset and progression of oxidation. The strong correlation between these parameters suggests that factors accelerating oxidation simultaneously shorten the lag phase and oxidation duration, highlighting their joint responsiveness to oxidative stress. The parameter β provides insight into the oxidation kinetics profile. Values of β greater than 1, indicate a sigmoidal curve, by a slow initial oxidation phase, followed by acceleration and eventual plateauing. The higher values observed suggest that oxidation does not follow a simple exponential trend but involves an initial resistance phase due to protective mechanisms, which, once overwhelmed, results in rapid oxidation. Variations in β across different conditions indicate that the oxidation process is influenced not only by the time delay (LagP) and the characteristic time (α) but also by other environmental factors. These factors affect the steepness of the oxidation curve, captured by β, and thereby define the nature of the oxidation response. The Weibull model is particularly appropriate to describe these kinetics as it integrates an initial delay (LagP), a flexible progression pattern (β), and a characteristic time scale (α).

### 3.3 Application of the method for evaluating interactions between αTOH and other antioxidants

Several antioxidants, including carnosic acid (CA, a diterpene), myricetin (Myr, a flavonoid), dieckol (Dl, a phlorotannin), and ascorbic acid (AA, also known as vitamin C) were evaluated in combination with αTOH. Two concentrations of αTOH in oil, 380 ppm and 1140 ppm, were selected and added to the oil before emulsification. These correspond to 0.2 μM (T0.2) and 0.6 μM (T0.6) of αTOH after dispersion in the final emulsion. The lower αTOH concentration (380 ppm, T0.2) reflects levels typically found in refined vegetable oils, while the higher concentration (1140 ppm, T0.6) represents those used in tocopherol-enriched food products. Notably, this upper concentration has been identified as a critical threshold beyond which prooxidant-induced mechanisms from tocopherols may become significant (Barouh et al., 2022). To evaluate the impact of both concentration and antioxidant ratio, three molar ratios of αTOH to the second AOX were tested: 1:0.3 (0.3x), 1:1 (1x), and 1:3 (3x). Moreover, since transition metals such as iron and copper are known to play a crucial role in oxidation pathways, particularly in accelerating hydroperoxide decomposition (Yamamoto and Niki, 1988) or by interacting with antioxidants through redox reactions (which can significantly impact the effectiveness of the antioxidant combination), their impact on antioxidant interactions was also investigated. For this purpose, the method was also extended to include ferrous iron to assess how transition metals impact antioxidant interactions. Following the evaluation of various concentrations, ferrous iron was added to the microplate well at 0.1 μM, as this level provided the most suitable oxidation kinetics in tung oil for assessing and ranking the effects of antioxidant combinations.

Varying the αTOH:AOX molar ratios can lead to different antioxidant response scenarios (Table 1). This representation facilitates the identification of the most effective AOX to combine with αTOH, as well as the determination of its optimal conditions in terms of concentration and ratio.

**Table 1:** Representative trends observed with increasing αTOH:AOX molar ratios.

Trends	Descriptions	Hypothesis	Illustration
T1	Linear dependence increases of AE and CF with increasing AOX ratios	Concentration-dependent – often resulting in synergistic interaction	
T2	Linear dependence increases of AE and CF up to a defined threshold with optimal concentration (ratio) of AOX	Concentration-dependent – often resulting in synergistic interaction up to a maximum for which prooxidant pathways may be observed.	
T3	A linear increase in AE is observed with no significant variation in CF as AOX increases.	Concentration-dependent - often resulting in additive interaction when CF is close to 1	
T4	Linear dependence decreases of AE and CF with increasing AOX ratios	Concentration-dependent – often resulting in prooxidant interaction	

#### 3.3.1 Assessment of combination efficiency in the absence of metal-induced oxidation

**Fig. 4:**
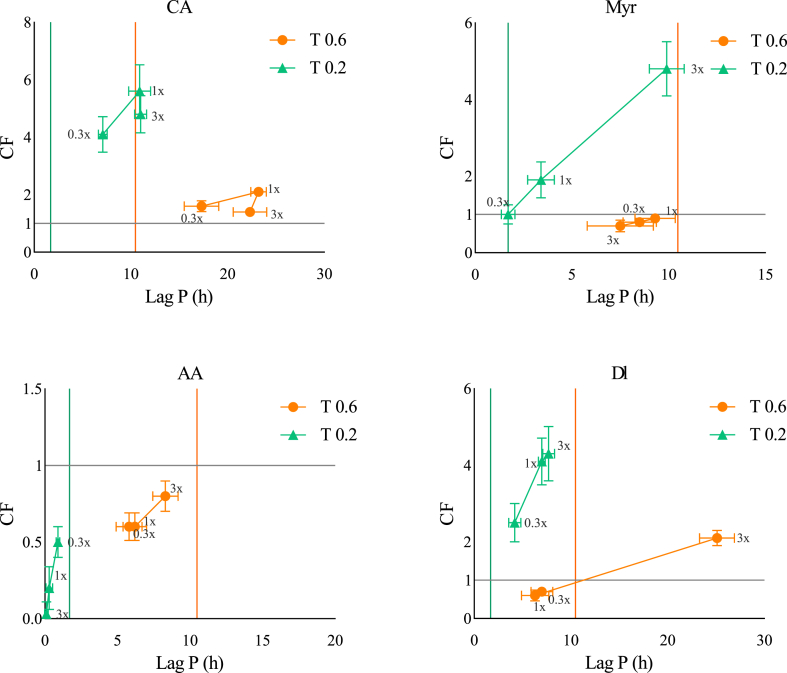
Effect of increasing αTOH: AOX ratios (0.3x (1:0.3), 1x (1:1), 3x (1:3)) on the *CF* and AE (lag phases) at two αTOH concentrations (0.2 and 0.6 μM in the emulsion). CA = carnosic acid, Myr = myricetin, AA = ascorbic acid, Dl = dieckol. Colored vertical lines indicate the LagP of αTOH at each specific concentration.

The CF of αTOH with CA, Myr, AA, and Dl, at two αTOH concentrations and three molar ratios (αTOH:AOX) are shown in Fig. 4. The Y-axis represents the CF while the X-axis corresponds to the AE based on LagP (h). The highest AE values were observed at T0.6 for αTOH:Dl and αTOH:CA at 1:3 and 1:1 ratio, respectively. In contrast, the highest CF values were observed at T0.2 for αTOH:CA and αTOH:Myr at 1:1 and 1:3 ratios, respectively. At the lower αTOH concentration (T0.2), CA, Myr, and Dl exhibited synergistic effects with αTOH in a concentration-dependent manner (**see trends T1 and T2**). Deciphering the mechanisms behind these synergistic effects remains challenging due to the multiplicity of potential pathways. For instance, CA or Myr may act synergistically with αTOH by regenerating its oxidized forms, a mechanism reported in various systems and attributed to their relatively low redox potentials (∼0.3V) (Bayram et al., 2023; Bayram and Decker, 2024; Lei et al., 2019; Hopia et al., 1996; Bouizgma et al., 2024). The results suggest that this regeneration effect appears more pronounced at higher concentrations. In the case of Dl, its eckol structure contributes to its high antioxidant activity in a concentration-dependent manner. Its structure may also trigger its ability to regenerate αTOH oxidized forms, although no study has yet confirmed this behaviour (Lim et al., 2024; Pyeon et al., 2021; Shibata et al., 2008). Interestingly, when the molar ratio of αTOH:CA or αTOH:Dl exceeds 1:1, AE no longer increases while CF decreases, (indicating the possible onset of prooxidant pathways (**see trend T2**). These prooxidant pathways may be attributed to the oxidized products of CA, such as quinones, which are unstable and susceptible to reduction (Masuda et al., 2002). Thus, an accumulation of quinones can obstruct the regeneration of αTOH. Regarding AA, its combination with αTOH exhibited primarily antagonistic effects, which became more pronounced with increasing AA concentration. This antagonism likely results from (i) unfavourable partitioning, as αTOH predominantly concentrates in the oil phase, and AA in the aqueous phase, thus limiting potential regeneration interactions, and (ii) the low reduction potential (∼0.3V) of AA, making it highly prooxidant (Njus et al., 2020).

At the highest αTOH concentration (T0.6), the pattern of interaction shifts slightly, underscoring the role of tocopherol concentration in modulating antioxidant or prooxidant pathways (Barouh et al., 2022). At this concentration, αTOH appears to promote prooxidant mechanisms more strongly, such as those induced by αTO° or αTO-OO° oxidation, or the reduction of hydroperoxide species by αTOH, among others. Within this context, both Dl and Myr tend to shift toward additive or even prooxidant interactions, except at the highest DI:αTOH molar ratios (1:3). In this particular case, an excess of Dl seems to favor synergistic interactions. Although likely influenced by multiple factors and reaction pathways, this higher Dl concentration may enhance its contact with αTOH and/or hydroperoxides at the oil–water interface, potentially mitigating the prooxidant effects of αTOH while promoting their positive interactions. Myr, at any tested concentration, seems to be ineffective in counterbalancing or decreasing the prooxidant alternative reactions of αTOH, as highlighted by a loss of its synergistic interactions with αTOH. It is possible that a higher Myr:αTOH ratio would be needed to restore synergistic effect on lipid oxidation. In contrast, a high concentration of αTOH does not alter the overall trends observed for CA and AA. For CA, the synergistic interaction is retained, with a maximum CF at a 1:1 molar ratio. For AA, the prooxidant behavior remains consistent. However, a noticeable decline in CF values was observed for CA at the higher αTOH concentration indicating a possible limitation in its capacity to maintain synergy at elevated tocopherol levels.

The main conclusions from the study are as follows:

- CA and αTOH: A synergistic interaction is observed between CA and αTOH, with the greatest synergy occurring at a 1:1 molar ratio. The highest CF factor is obtained at lower αTOH concentrations.
- AA and αTOH: A consistently prooxidant interaction is observed between ascorbic acid (AA) and αTOH, regardless of concentration or molar ratio tested.
- Myr and αTOH: Myricetin (Myr) exhibits a concentration-dependent synergistic interaction with αTOH at the lower tocopherol concentration, with the strongest effect at a 1:3 molar ratio. However, at the higher αTOH concentration, the interaction becomes either additive or prooxidant.
- Dl and αTOH: A concentration-dependent synergistic interaction is observed between Dl and αTOH at the lower tocopherol level. At the higher αTOH concentration, a greater Dl concentration is required to maintain a synergistic interaction.

#### 3.3.2 Assessment of combination efficiency in the presence of metal-induced oxidation

**Fig. 5:**
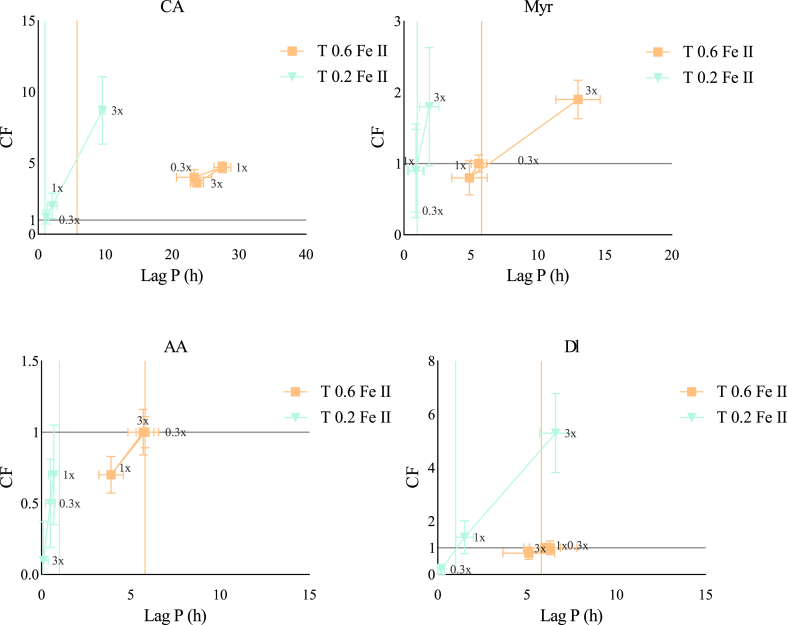
Effect of ferrous ion addition (0.1 μM) on the CF and lag phases of antioxidant combinations with two different concentrations of αTOH (0.2 and 0.6 μM in emulsion).

The CF of αTOH with CA, Dl, Myr, and AA in the presence of ferrous ions (0.1 μM) was evaluated using the same methodology as previously described (Fig. 5). As expected, the transition metal (Fe^2+^) significantly accelerated the oxidation kinetics. At T0.6, the highest AE values were observed for the αTOH:CA and αTOH:Myr combinations at 1:1 and 1:3 ratios, respectively. While at T0.2, the highest CF values were achieved with αTOH:CA and αTOH:Dl at a 1:3 ratio. At the lower αTOH concentration (T0.2), the addition of CA and Dl demonstrated synergistic interactions with both AE and CF increasing linearly with concentration (see trend T1). These findings suggest that the beneficial combination effect is more pronounced at higher antioxidant:αTOH ratios. In contrast combinations with Myr, primarily exhibited additive effects without any concentration-dependent trend, indicating that iron plays a significant role in modulating the synergistic potential of αTOH:Myr pair (Fig. 5). As for AA, antagonist interactions were again observed, consistent with results in the absence of ferrous ions (Fig. 5), thereby confirming the dominant prooxidant pathways of AA such as recycling ferric to ferrous ions (Gęgotek et al., 2023) and/or its limited positive interactions with αTOH.

At the highest αTOH concentration (T 0.6), the outcomes shifted slightly once again. At its highest concentration, Myr regained a synergistic interaction with αTOH, while the 1:1 αTOH and CA molar ratio resulted in the maximum threshold in CF. Dl exhibited additive effects in combination with αTOH, while AA failed to provide a positive CF, confirming its unsatisfactory combination with αTOH.

The addition of ferrous ions triggers additional dominant mechanisms that can significantly influence the antioxidant performance in the different combinations (Schaich, 1992) making the mechanistic hypothesis more challenging. Accordingly, at the lowest αTOH concentration (T0.2), an excess of AOX is recommended to enhance both CF and AE under conditions involving prooxidant metals.

Thus, the main conclusions resulting from the combination effect of αTOH:AOX in the presence of metal-induced oxidation are:

- CA and αTOH: Synergistic interactions are maintained in a concentration-dependent of CA at T0.2 At higher αTOH concentration (T0.6), maximum synergy is achieved at a 1:1 molar ratio.
- AA and αTOH: AA consistently exhibits antagonistic interactions with αTOH, mirroring its behavior in the absence of ferrous ions.
- Myr and αTOH: At T0.2, the association primarily results in additive effects. However, at T0.6, synergistic interactions are restored at the highest αTOH:Myr ratio of 1:3.
- Dl and αTOH: At T0.2, a high concentration of Dl is required to maintain synergistic interactions with αTOH whereas, at T0.6, the interactions are predominantly additive.

#### 3.3.3 Interpretation of oxidation propagation kinetics using α- and β-factors

**Fig. 6:**
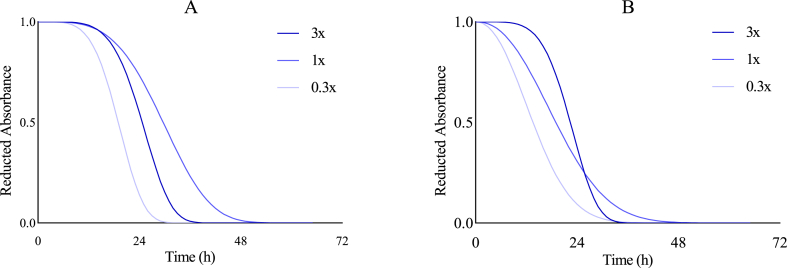
Oxidation Kinetics resulting from the combination of carnosic acid (CA) and αTOH at 0.2 μM, with increasing CA:αTOH ratios (0.3x (1:0.3), 1x (1:1), 3x (1:3)), in the absence (A) and presence (B) of metal-induced oxidation (0.1 μM Fe^2+^).

In addition to CF and AE (both derived from LagP and primarily focused on interpreting the initiation phase), α- and β-factors calculated from Weibull modeling can be used to describe the propagation phase of oxidation kinetics. In this section, we focus on CA, given its high AE and CF values which make it a promising candidate for studying synergy with αTOH. However, CA exhibits a more complex behavior with a threshold value of LagP at a certain concentration, which warrants a deeper analysis using Weibull parameters. In fact, in the CA and αTOH combination, despite similar LagP, significantly different α values are obtained (Fig. 6A and Table 2). A higher α indicates a more favorable interaction in delaying the kinetics during the propagation phase of oxidation. In contrast, similar α values can be obtained, while significantly different LagP are observed (Fig. 6B and Table 2). This example under iron induced oxidation, likely because it significantly accelerates the propagation of oxidation, while drastically reducing the initiation phase. Regarding the β-factor, which characterizes the shape of the kinetic curve and highlights the speed of the propagation phase, different β-factors are obtained for the same α values when comparing the 3x and 1x combinations (Fig. 6B). This indicates that prooxidative pathways induced by ferrous ion are less suppressed, and that the propagation phase proceeds more slowly with an equimolar ratio between the two antioxidants, despite a shorter initiation phase. This observation supports the conclusion that the most effective synergistic interactions occurs at an equimolar ratio between CA and αTOH (T0.2). These examples illustrate how, in addition to CF and AE, this method can be applied on a case-by-case basis to thoroughly evaluate the combined antioxidant effects throughout the entire oxidation process.

**Table 2:** Determination of the Weibull model kinetic parameters (α- and β) and lag phase (LagP) under various oxidation conditions for the combination of carnosic acid (CA) and αTOH at 0.2 μM (Complete data for the other tested combinations are available in the **supplementary data)**.

Ratio αTOH:CA	Condition of oxidation	LagP (h)	α (h)	β	RMSE
1:3 (3x)	Non-induced	11.0 ± 0.6^a^	26.5 ± 0.3^a^	5.1 ± 0.3^a^	0.05
1:1 (1x)	Non-induced	10.9 ± 1.1^a^	32.6 ± 0.6^b^	3.8 ± 0.3^b^	0.08
1:0.3 (0.3x)	Non-induced	7.1 ± 0.4^b^	20.6 ± 0.2^c^	4.5 ± 0.2^a, b^	0.04
1:3 (3x)	Metal-induced oxidation	9.6 ± 0.4^c^	24.1 ± 1.1^d^	5.0 ± 0.4^c^	0.03
1:1 (1x)	Metal-induced oxidation	2.1 ± 0.7^d, e^	22.3 ± 0.7^d^	2.3 ± 0.3^d^	0.15
1:0.3 (0.3x)	Metal-induced oxidation	1.2 ± 0.3^e^	16.1 ± 0.4^e^	2.2 ± 0.3^d^	0.11

Parameter value ± confidence interval (*p* = 0.05).

RMSE: Root mean square error of reduced absorbance ($A^{*}$) between experimental and simulated data.

Different superscript letters (a-e) in the same column indicate statistically significant differences (*p* < 0.05).

## 4 Conclusions

Methods for evaluating synergistic interactions between antioxidants in lipid oxidation are scarce and often limited by significant drawbacks in existing assays. To address this gap, we developed a spectrophotometric assay coupled with mathematical modeling (the Weibull interaction modeling CAT: WIM-CAT), which offers a robust analytical approach. This method avoids the use of azo initiators or buffers in order to prevent any pro or antioxidant pathways arising from the presence of such compounds. Furthermore, the assay can be conducted both in the absence and presence of iron-induced oxidation, thereby highlighting the impact of transition metals on the efficiency of antioxidant combinations. The use of Weibull modeling provides a better understanding of oxidation kinetics and enables the identification of key parameters (LagP, α, β) critical for the assessment of antioxidant effectiveness. This study reveals the complex interactions between αTOH and various antioxidants, demonstrating that the effectiveness of these interactions is highly concentration-dependent. Synergistic effects between αTOH and antioxidants, such as CA, Dl, and Myr, can significantly improve the oxidative stability of lipid emulsions, with concentration-dependent synergy observed in multiple combinations. Furthermore, while interacts synergistically with some antioxidants, AA consistently antagonizes αTOH's antioxidant activity. Additionally, the presence of ferrous ions influences the mechanisms involved between antioxidants and highlights the role of metal ions in modulating antioxidant mechanisms. CA and Dl emerge as particularly promising candidates for combination with αTOH due to their high AE and CF. The findings offer valuable insights into the mechanisms of interaction between antioxidants and αTOH. Overall, this rapid method, requiring only standard spectrophotometric equipment, lays a foundation for the development of more effective antioxidant formulations facilitating the assessment of medium effects or combinations with other natural antioxidants.

## CRediT authorship contribution statement

**Camille Robichon:** Conceptualization, Methodology, Investigation, Formal analysis, Writing – original draft, Software. **Pierre Villeneuve:** Conceptualization, Supervision, Resources, Writing – review & editing. **Philippe Bohuon:** Software, Formal analysis, Writing – review & editing. **Bruno Baréa:** Resources, Writing – review & editing. **Nathalie Barouh:** Investigation, Writing – review & editing. **Francis Courtois:** Writing – review & editing. **Frédéric Fine:** Funding acquisition, Writing – review & editing. **Erwann Durand:** Conceptualization, Supervision, Resources, Writing – review & editing.

## Ethics statement

No ethics statement is required for the research presented in this study since neither procedures nor raw materials involved animals or animal-derived products.

## Declaration of competing interest

The authors declare that they have no known competing financial interests or personal relationships that could have appeared to influence the work reported in this paper.

## Data Availability

Data will be made available on request.

## References

[bib1] Amorati R., Valgimigli L. (2015). Advantages and limitations of common testing methods for antioxidants. Free Radic. Res..

[bib2] Azzi A. (2018). Many tocopherols, one vitamin E. Mol. Aspect. Med..

[bib3] Barbieri J.B., Goltz C., Batistão Cavalheiro F., Theodoro Toci A., Igarashi-Mafra L., Mafra M.R. (2020). Deep eutectic solvents applied in the extraction and stabilization of rosemary (Rosmarinus officinalis L.) phenolic compounds. Ind. Crops Prod..

[bib4] Barouh N., Bourlieu-Lacanal C., Figueroa-Espinoza M.C., Durand E., Villeneuve P. (2022). Tocopherols as antioxidants in lipid-based systems: the combination of chemical and physicochemical interactions determines their efficiency. Compr. Rev. Food Sci. Food Saf..

[bib5] Bayram I., Decker E.A. (2023). Underlying mechanisms of synergistic antioxidant interactions during lipid oxidation. Trends Food Sci. Technol..

[bib6] Bayram I., Decker E.A. (2024). Analysis of the mechanism of antioxidant synergism between α-tocopherol and myricetin in bulk oil. J. Am. Oil Chem. Soc..

[bib7] Bayram I., Laze A., Decker E.A. (2023). Synergistic mechanisms of interactions between myricetin or taxifolin with α-tocopherol in oil-in-water emulsions. J. Agric. Food Chem..

[bib8] Bayram I., Parra-Escudero C., Decker E.A., Lu J. (2024). Mathematical modeling of alpha-tocopherol early degradation kinetics to predict the shelf-life of bulk oils. J. Agric. Food Chem..

[bib9] Becker E.M., Ntouma G., Skibsted L.H. (2007). Synergism and antagonism between quercetin and other chain-breaking antioxidants in lipid systems of increasing structural organisation. Food Chem..

[bib10] Bouizgma K., Rabbah N., Abbas Z., Abourriche A. (2024). Enhancing the stability of soybean oil: using endogenous phenolic compounds additive and carnosic acid plus carnosol. ACS Food Sci. Technol..

[bib11] Daoud S., Bou-Maroun E., Waschatko G., Cayot P. (2021). Lipid oxidation in oil-in-water emulsions: iron complexation by buffer ions and transfer on the interface as a possible mechanism. Food Chem..

[bib12] Decker E.A., McClements D.J., Bourlieu-Lacanal C., Durand E., Figueroa-Espinoza M.C., Lecomte J., Villeneuve P. (2017). Hurdles in predicting antioxidant efficacy in oil-in-water emulsions. Trends Food Sci. Technol..

[bib13] Delgado A.M., Isaac A., García A., Cambero J.A. (2020). Contribution of tocols to food sensorial properties, stability, and overall quality. J. Food Qual..

[bib14] Durand E., Laguerre M., Bourlieu-Lacanal C., Lecomte J., Villeneuve P. (2025). Navigating the complexity of lipid oxidation and antioxidation: a review of evaluation methods and emerging approaches. Prog. Lipid Res..

[bib15] Evans H.M., Bishop K.S. (1922). On the existence of a hitherto unrecognized dietary factor essential for reproduction. Science.

[bib16] Gęgotek A., Skrzydlewska E., Litwack G. (2023).

[bib17] Gordon M.H., Decker E.A. (2010). Oxidation in Foods and Beverages and Antioxidant Applications.

[bib18] Goujot D., Cuvelier M.-E., Soto P., Courtois F. (2019). A stoichio-kinetic model for a DPPH∙-ferulic acid reaction. Talanta.

[bib19] Hennebelle M., Villeneuve P., Durand E., Lecomte J., Van Duynhoven J., Meynier A., Yesiltas B., Jacobsen C., Berton-Carabin C. (2024). Lipid oxidation in emulsions: new insights from the past two decades. Prog. Lipid Res..

[bib20] Hopia A.I., Huang S.-W., Schwarz K., German J.B., Frankel E.N. (1996). Effect of different lipid systems on antioxidant activity of rosemary constituents carnosol and carnosic acid with and without α-tocopherol. J. Agric. Food Chem..

[bib21] Kittipongpittaya K., Panya A., Phonsatta N., Decker E.A. (2016). Effects of environmental pH on antioxidant interactions between rosmarinic acid and α-tocopherol in oil-in-water emulsions. J. Agric. Food Chem..

[bib22] Laguerre M., Lecomte J., Villeneuve P. (2007). Evaluation of the ability of antioxidants to counteract lipid oxidation: existing methods, new trends and challenges. Prog. Lipid Res..

[bib23] Laguerre M., López-Giraldo L.J., Lecomte J., Baréa B., Cambon E., Tchobo P.F., Barouh N., Villeneuve P. (2008). Conjugated autoxidizable triene (CAT) assay: a novel spectrophotometric method for determination of antioxidant capacity using triacylglycerol as ultraviolet probe. Anal. Biochem..

[bib24] Lei C., Tang X., Chen M., Chen H., Yu S. (2019). Alpha-tocopherol-based microemulsion improving the stability of carnosic acid and its electrochemical analysis of antioxidant activity. Colloids Surf. A Physicochem. Eng. Asp..

[bib25] Lim S.-B., Lee J., Yang Y.-H., Son H., Yoo H.Y., Han J.-A. (2024). Development of a novel functional jelly with dieckol-rich extract from *Eisenia bicyclis*: physicochemical, antioxidant, and sensory characterization. Food Chem. X.

[bib26] Masuda T., Inaba Y., Maekawa T., Takeda Y., Tamura H., Yamaguchi H. (2002). Recovery mechanism of the antioxidant activity from carnosic acid quinone, an oxidized sage and rosemary antioxidant. J. Agric. Food Chem..

[bib27] Njus D., Kelley P.M., Tu Y.-J., Schlegel H.B. (2020). Ascorbic acid: the chemistry underlying its antioxidant properties. Free Radic. Biol. Med..

[bib28] Panya A., Temthawee W., Phonsatta N., Charoensuk D., Deetae P., Visessanguan W., Decker E.A. (2015). Apolar radical initiated conjugated autoxidizable triene (ApoCAT) assay: effects of oxidant locations on antioxidant capacities and interactions. J. Agric. Food Chem..

[bib29] Parra-Escudero C., Bayram I., Decker E.A., Singh S., Corvalan C.M., Lu J. (2025). A machine learning-guided modeling approach to the kinetics of α-tocopherol and myricetin synergism in bulk oil oxidation. Food Chem..

[bib30] Phonsatta N., Grajeda-Iglesias C., Figueroa-Espinoza M.C., Baréa B., Lecomte J., Visessanguan W., Durand E., Villeneuve P., Tapingkae W., Panya A. (2020). Investigation on the double cut-off phenomenon observed in protocatechuic acid and its alkyl esters under various CAT-based assays. J. Agric. Food Chem..

[bib31] Pyeon D.-B., Lee S.-E., Yoon J.-W., Park H.-J., Park C.-O., Kim S.-H., Oh S.-H., Lee D.-G., Kim E.-Y., Park S.-P. (2021). The antioxidant dieckol reduces damage of oxidative stress-exposed porcine oocytes and enhances subsequent parthenotes embryo development. Mol. Reprod. Dev..

[bib32] Schaich K.M. (1992). Metals and lipid oxidation. Contemporary issues. Lipids.

[bib33] Schaich K.M. (2024). Epoxides: an underestimated lipid oxidation product. Free Radic. Res..

[bib34] Schroën K., Berton-Carabin C.C. (2022). A unifying approach to lipid oxidation in emulsions: modeling and experimental validation. Food Res. Int..

[bib35] Shahidi F., Hossain A. (2022). Role of lipids in food flavor generation. Molecules.

[bib36] Shibata T., Ishimaru K., Kawaguchi S., Yoshikawa H., Hama Y. (2008). Antioxidant activities of phlorotannins isolated from Japanese Laminariaceae. J. Appl. Phycol..

[bib37] Tang L., Cao M., Liao C., Liu R., Chang M., Wang X. (2023). Migration of tocopherols from the oil phase to the oil–water interface using phospholipids improved the oxidative stability of O/W emulsions. Food Chem..

[bib38] van Boekel M.A.J.S. (2002). On the use of the Weibull model to describe thermal inactivation of microbial vegetative cells. Int. J. Food Microbiol..

[bib39] Vieira S.A., Zhang G., Decker E.A. (2017). Biological implications of lipid oxidation products. J. Am. Oil Chem. Soc..

[bib40] Yamamoto K., Niki E. (1988). Interaction of α-tocopherol with iron: antioxidant and prooxidant effects of α-tocopherol in the oxidation of lipids in aqueous dispersions in the presence of iron. Biochim. Biophys. Acta Lipids Lipid. Metabol..

[bib41] Yoshimura Y., Matsuzaki Y., Watanabe T., Uchiyama K., Ohsawa K., Imaeda K. (1992). Effects of buffer solutions and chelators on the generation of hydroxyl radical and the lipid peroxidation in the Fenton reaction system. J. Clin. Biochem. Nutr..

[bib42] Zhang C., Wang Y., Yu Z., Xu Y., Guo Y., Liu R., Chang M., Wang X. (2024). Enhancing the oxidation stability and bioaccessibility of algal oil emulsion by using tocopherol and chlorogenic acid. Food Biosci..

