# Supplementary material for: Unveiling synergistic antioxidant combinations for α-tocopherol in emulsions: A spectrophotometric-mathematical approach
Source: Curr Res Food Sci. 2025 Jul 2;11:101134. doi: 10.1016/j.crfs.2025.101134 (PMC12273494; doi:10.1016/j.crfs.2025.101134)
Supplement: Multimedia component 1 [file mmc1.docx]

|  | [α-TOCO] | Ratio | α | stat p<0.05 | β | stat p<0.05 | Tlag (h) | stat p<0.05 | RMSE | R² |
| --- | --- | --- | --- | --- | --- | --- | --- | --- | --- | --- |
| CA | 0.6 µM | (1:3) | 43.8 ± 0.2 | a | 5.6 ± 0.7 | c | 22.3 ± 1.7 | d | 0.11 | 0.93 |
|  | 0.6 µM | (1:1) | 43.3 ± 0.1 | a | 6.1 ± 0.3 | c | 23.2 ± 0.8 | d | 0.05 | 0.98 |
|  | 0.6 µM | (1:0.3) | 38.7 ± 0.2 | b | 4.9 ± 0.6 | c | 17.3 ± 1.8 | e | 0.11 | 0.93 |
|  | 0.2 µM | (1:3) | 26.5 ± 1.7 | f | 5.1 ± 0.3 | i | 11.0 ± 0.6 | k | 0.05 | 0.99 |
|  | 0.2 µM | (1:1) | 32.6 ± 0.4 | g | 3.8 ± 0.3 | j | 10.9 ± 1.1 | kl | 0.08 | 0.97 |
|  | 0.2 µM | (1:0.3) | 20.6 ± 0.4 | h | 4.5 ± 0.2 | i | 7.1 ± 0.4 | l | 0.04 | 0.99 |
| CA + Fe II | 0.6 µM | (1:3) | 44.9 ± 0.1 | a | 5.8 ± 0.4 | c | 23.7 ± 1.0 | d | 0.06 | 0.98 |
|  | 0.6 µM | (1:1) | 50.9 ± 0.1 | b | 5.7 ± 0.4 | c | 27.5 ± 1.3 | e | 0.07 | 0.96 |
|  | 0.6 µM | (1:0.3) | 45.7 ± 0.1 | a | 5.1 ± 0.7 | c | 23.3 ± 2.6 | de | 0.13 | 0.89 |
|  | 0.2 µM | (1:3) | 24.1 ± 0.6 | f | 5.0 ± 0.2 | h | 9.6 ± 0.4 | j | 0.03 | 0.99 |
|  | 0.2 µM | (1:1) | 22.3 ± 0.5 | f | 2.3 ± 0.4 | i | 2.1 ± 0.8 | k | 0.15 | 0.88 |
|  | 0.2 µM | (1:0.3) | 16.1 ± 0.3 | g | 2.2 ± 0.3 | i | 1.2 ± 0.3 | k | 0.11 | 0.93 |
| MYR | 0.6 µM | (1:3) | 39.2 ± 0.1 | a | 2.6 ± 0.4 | c | 7.5 ± 1.7 | e | 0.19 | 0.82 |
|  | 0.6 µM | (1:1) | 24.2 ± 0.0 | b | 4.8 ± 0.5 | d | 9.3 ± 1.0 | e | 0.08 | 0.96 |
|  | 0.6 µM | (1:0.3) | 24.2 ± 0.0 | b | 4.4 ± 0.4 | d | 8.5 ± 0.9 | e | 0.07 | 0.97 |
|  | 0.2 µM | (1:3) | 23.6 ± 0.8 | f | 5.4 ± 0.6 | h | 9.9 ± 0.9 | j | 0.07 | 0.97 |
|  | 0.2 µM | (1:1) | 17.6 ± 0.7 | g | 3.2 ± 0.3 | i | 3.4 ± 0.7 | k | 0.07 | 0.96 |
|  | 0.2 µM | (1:0.3) | 17.8 ± 0.5 | g | 2.5 ± 0.3 | i | 1.7 ± 0.4 | k | 0.08 | 0.95 |
| MYR + Fe II | 0.6 µM | (1:3) | 27.4 ± 0.5 | a | 6.1 ± 1.0 | d | 13.0 ± 1.6 | e | 0.12 | 0.94 |
|  | 0.6 µM | (1:1) | 16.2 ± 0.1 | b | 4.2 ± 0.7 | d | 4.9 ± 1.3 | f | 0.10 | 0.95 |
|  | 0.6 µM | (1:0.3) | 17.6 ± 0.1 | c | 4.4 ± 0.4 | d | 5.6 ± 0.6 | f | 0.05 | 0.98 |
|  | 0.2 µM | (1:3) | 14.2 ± 0.7 | g | 2.8 ± 0.4 | h | 1.9 ± 0.7 | i | 0.11 | 0.93 |
|  | 0.2 µM | (1:1) | 15.8 ± 0.4 | g | 2.1 ± 0.4 | h | 0.9 ± 0.6 | i | 0.14 | 0.89 |
|  | 0.2 µM | (1:0.3) | 14.4 ± 0.4 | g | 2.2 ± 0.3 | h | 0.9 ± 0.5 | i | 0.12 | 0.91 |
| D | 0.6 µM | (1:3) | 50.6 ± 0.1 | a | 5.0 ± 0.5 | c | 25.1 ± 1.8 | d | 0.10 | 0.90 |
|  | 0.6 µM | (1:1) | 22.1 ± 0.0 | b | 3.8 ± 0.6 | c | 6.3 ± 1.4 | e | 0.12 | 0.92 |
|  | 0.6 µM | (1:0.3) | 22.7 ± 0.0 | b | 4.0 ± 0.5 | c | 7.0 ± 1.1 | e | 0.09 | 0.96 |
|  | 0.2 µM | (1:3) | 18.1 ± 0.4 | f | 5.9 ± 0.5 | h | 7.7 ± 0.6 | j | 0.05 | 0.98 |
|  | 0.2 µM | (1:1) | 17.0 ± 0.5 | g | 5.7 ± 0.3 | h | 7.0 ± 0.4 | j | 0.03 | 0.99 |
|  | 0.2 µM | (1:0.3) | 16.5 ± 0.8 | g | 3.8 ± 0.4 | i | 4.2 ± 0.6 | f | 0.06 | 0.98 |
| D + Fe II | 0.6 µM | (1:3) | 20.8 ± 0.0 | a | 3.5 ± 0.6 | d | 5.1 ± 1.4 | e | 0.12 | 0.92 |
|  | 0.6 µM | (1:1) | 16.3 ± 0.1 | b | 5.0 ± 0.7 | d | 6.3 ± 1.5 | e | 0.09 | 0.96 |
|  | 0.6 µM | (1:0.3) | 19.0 ± 0.2 | c | 4.3 ± 0.5 | d | 6.0 ± 0.9 | e | 0.08 | 0.97 |
|  | 0.2 µM | (1:3) | 14.1 ± 0.2 | f | 6.8 ± 0.7 | h | 6.6 ± 0.9 | k | 0.05 | 0.98 |
|  | 0.2 µM | (1:1) | 10.9 ± 0.6 | g | 3.1 ± 0.5 | i | 1.5 ± 0.5 | l | 0.10 | 0.95 |
|  | 0.2 µM | (1:0.3) | 9.5 ± 0.5 | g | 1.9 ± 0.3 | j | 0.2 ± 0.2 | l | 0.10 | 0.94 |
| AA | 0.6 µM | (1:3) | 23.3 ± 0.0 | a | 4.5 ± 0.4 | c | 8.3 ± 0.9 | e | 0.07 | 0.97 |
|  | 0.6 µM | (1:1) | 21.6 ± 0.1 | b | 3.8 ± 0.4 | cd | 6.2 ± 0.8 | e | 0.08 | 0.97 |
|  | 0.6 µM | (1:0.3) | 25.1 ± 0.1 | a | 3.1 ± 0.4 | d | 5.8 ± 0.9 | e | 0.12 | 0.93 |
|  | 0.2 µM | (1:3) | 9.1 ± 1.0 | f | 1.6 ± 0.2 | h | 0.1 ± 0.1 | k | 0.11 | 0.92 |
|  | 0.2 µM | (1:1) | 11.7 ± 0.5 | g | 1.9 ± 0.3 | hi | 0.3 ± 0.2 | kl | 0.10 | 0.94 |
|  | 0.2 µM | (1:0.3) | 11.8 ± 0.5 | g | 2.6 ± 0.2 | i | 0.9 ± 0.1 | l | 0.05 | 0.98 |
| AA + Fe II | 0.6 µM | (1:3) | 17.3 ± 0.2 | a | 4.6 ± 0.3 | d | 5.8 ± 0.5 | f | 0.04 | 0.99 |
|  | 0.6 µM | (1:1) | 20.3 ± 0.2 | b | 3.1 ± 0.3 | e | 3.9 ± 0.7 | f | 0.09 | 0.95 |
|  | 0.6 µM | (1:0.3) | 23.0 ± 0.1 | c | 3.4 ± 0.3 | de | 5.7 ± 0.9 | fg | 0.08 | 0.97 |
|  | 0.2 µM | (1:3) | 7.9 ± 0.7 | h | 1.4 ± 0.2 | k | 0.1 ± 0.3 | m | 0.11 | 0.91 |
|  | 0.2 µM | (1:1) | 15.4 ± 0.6 | i | 2.1 ± 0.3 | l | 0.7 ± 0.3 | m | 0.10 | 0.94 |
|  | 0.2 µM | (1:0.3) | 15.4 ± 0.6 | ji | 2.0 ± 0.2 | kl | 0.5 ± 0.3 | m | 0.09 | 0.95 |
